# Optimization of Supercritical Fluid Extraction of Total Alkaloids, Peimisine, Peimine and Peiminine from the Bulb of *Fritillaria thunbergii* Miq, and Evaluation of Antioxidant Activities of the Extracts

**DOI:** 10.3390/ma9070524

**Published:** 2016-06-29

**Authors:** Xiao Ruan, Li Yang, Wen-Xia Cui, Men-Xing Zhang, Zhao-Hui Li, Ben Liu, Qiang Wang

**Affiliations:** Ningbo Institute of Technology, Zhejiang University, Ningbo 315100, China; ruanxiao@nit.net.cn (X.R.); yangli0817@nit.net.cn (L.Y.); cuiwenxiao@163.com (W.-X.C.); zhangmenxing@163.com (M.-X.Z.); maylizhaohui@163.com (Z.-H.L.); liuben@nit.net.cn (B.L.)

**Keywords:** *Fritillaria thunbergii* Miq, supercritical fluid extraction, central composite design, total alkaloids, peimisine, peimine, peiminine, antioxidant capacity

## Abstract

Supercritical fluid extraction (SFE) was used to extract total alkaloids, peimisine, peimine and peiminine from the bulb of *Fritillaria thunbergii* Miq. The antioxidant capacity of the extracts was evaluated by DPPH radical scavenging activity (DPPH-RSA), ABTS radical scavenging activity (ABTS-RSA) and ferric reducing capacity (FRAP) assay. A central composite design (CCD) with four variables and five levels was employed for optimization of process parameters, and response surface plots were constructed in accordance with a second order polynomial model. Under optimal conditions of 3.0 h, 60.4 °C, 26.5 MPa and 89.3% ethanol, the highest yields were predicted to be 3.8 mg/g for total alkaloids, 0.5 mg/g for peimisine, 1.3 mg/g for peimine and 1.3 mg/g for peiminine, and the antioxidant capacity of extracts displayed EC_50, DPPH_ value of 5.5 mg/mL, EC_50, ABTS_ value of 0.3 mg/mL and FRAP value of 118.2 mg ascorbic acid equivalent (AAE)/100 g.

## 1. Introduction

**Figure 1:**
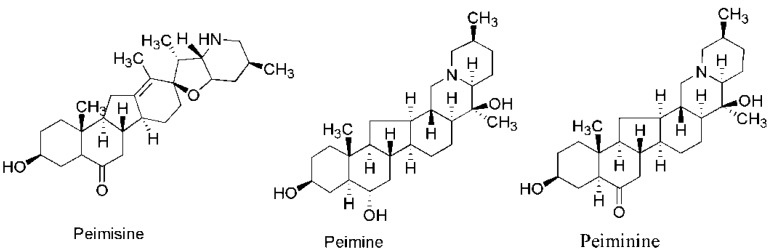
Chemical structure of peimisine, peimine and peiminine.

*Fritillaria* is a genus of 130–165 species [1,2] within the monocot family Liliaceae, and is native to temperate regions of the Northern Hemisphere [3]. The bulbs of *Fritillaria* species growing in China have been used as antitussive and expectorant herbs in Traditional Chinese Medicine (TCM) for more than 200 years, *Fritillaria thunbergii* Miq. (Chinese name Zhe Beimu) being the first one from genus *Fritillaria* [4]. Alkaloids, as the main active ingredients, contribute to the antitussive and expectorant function and they are usually extracted by classical solvent extraction [5,6]. According to Chinese Pharmacopeia (2010 edition), the content of peimine and peiminine in the bulb of *F.* *Thunbergii* extracted by the CHCl_3_/CH_3_OH = 4:1 and analyzed by HPLC must be higher than 0.1% for medical use (chemical structure of peimisine, peimine, and peiminine see Figure 1) [7]. However, solvent extraction is time-consuming and also causes some damages to the environment and health.

Supercritical carbon dioxide (SC-CO_2_) extraction is of great interest as a cost effective and environmentally friendly method for extracting useful components [8]. Supercritical fluid extraction (SFE) possesses several advantages such as good selectivity, environmental safety, less or no use of organic solvents, and higher speed [9]. Regarding the extraction of alkaloids, SC-CO_2_ has received considerable attention [10,11,12]. However, the SFE of alkaloids from the bulb of *F. Thunbergii* has not been disclosed in the literature so far. As known, one drawback of SC-CO_2_ is the non-polar nature of this solvent, but this problem can be resolved by the addition of a co-solvent such as ethanol, which is also classified as a natural or bio-derived solvent [13]. In addition, one of the main aspects under careful consideration in SFE is the extraction efficiency. The optimization of various variables influencing the SFE extractions could significantly enhance the recovery or extraction yield of a target compound [14]. A central composite design (CCD) based on response surface methodology (RSM) is an effective mathematical and statistical tool for performing, improving and optimizing the independent factors that influence response in a given set of experiments. It defines not only the effect of independent variables, but also their interaction effects [15].

The role of antioxidants in the maintenance of health and prevention of disorders and diseases has received much attention. Above all, the action and effects of natural antioxidants contained in foods have been the subjects of extensive studies [16,17,18]. The activity of antioxidants is usually measured by DPPH-RSA, ABTS-RSA, FRAP assay and CUPRAC (cupric reducing antioxidant capacity) assay [19,20,21]. Since the antioxidant activity of *F. thunbergii* bulbs has not been critically evaluated, its antioxidant activity was measured using three different methods (DPPH-RSA, ABTS-RSA and FRAP assays) to ascertain their potential as functional foods and functional ingredients in this paper.

In this study, different combinations of extraction time, temperature, pressure, and ethanol concentration as modifier were developed to an optimal SFE of alkaloids from the bulb of *F.* *thunbergii*, to evaluate the antioxidant activity of the total extract.

## 2. Results

### 2.1. UPLC Chromatogram

**Figure 2:**
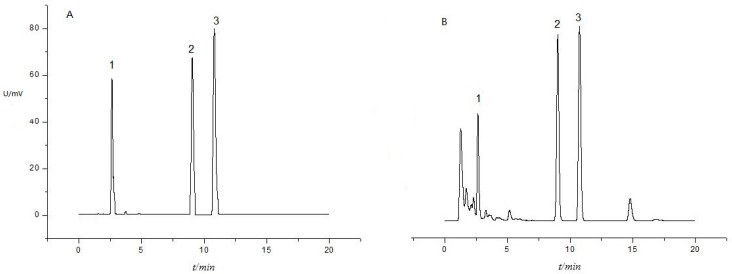
UPLC chromatogram obtained by supercritical CO_2_, 90% ethanol + 10% H_2_O (*v*/*v*) from a fix amount of cosolvent of 0.4 mL/min, at 25 MPa, 60 °C and an extraction time of 2 h: (**A**) standards; and (**B**) the extract of the bulb (1: peimisine (MW,427.3); 2: peimine (MW, 431.6); and 3: peiminine (MW, 429.6)).

Typical UPLC chromatogram of SFE extract of *F. thunbergii* bulb purified by solid-phase extraction column is shown in Figure 2B. Based on the available standards of peimisine, peimine and peiminine, it was possible to identify the peaks with retention times of 2.6, 9.1 and 10.8 min, respectively.

### 2.2. Optimization Strategy

Since various factors could potentially affect the extraction process, optimization of experimental conditions represented a critical step in the development of a SFE method. The effects of four independent process variables including extraction time (X_1_: 1.5–3.5 h), temperature (X_2_: 45–65 °C), pressure (X_3_: 10–30 MPa) and co-solvent concentration (X_4_: ethanol-water ratio, 80%–100%) were investigated during SFE of *F*. *Thunbergii* bulb. The four dependent responses of interest were related to total alkaloids, peimisine, peimine and peiminine yields. The experimental design and corresponding response data are presented in Table 1. As shown, the yields of total alkaloids, peimisine, peimine and peiminine varied from 1.8 to 3.7 mg/g, 0.04 to 0.5 mg/g, 0.3 to 1.2 mg/g and 0.2 to 1.2 mg/g, respectively, indicating that the extraction yields were highly influenced by extraction condition and that optimization was essential.

The mathematical model describing the extraction yield of total alkaloids (Y_1_, mg/g), peimisine (Y_2_, mg/g), peimine (Y_3_, mg/g) and peiminine (Y_4_, mg/g) as functions of the coded independent variables in the selected ranges was given by the following second-order polynomial equations, respectively.

(1) $$\begin{array}{l} \;Y_{1}(\mathrm{mg}/g)=3.47+0.33X_{1}+0.12X_{2}+0.28X_{3}+0.43X_{4}-0.18{X_{1}}^{2}-0.56{X_{2}}^{2}-0.14{X_{3}}^{2}-0.44{X_{4}}^{2}\\ +0.000625X_{1}X_{2}-0.037X_{1}X_{3}-0.066X_{1}X_{4}+0.019X_{2}X_{3}+0.023X_{2}X_{4}-0.049X_{3}X_{4} \end{array}$$

(2) $$\begin{array}{l} \;Y_{2}(\mathrm{mg}/g)=0.42+0.01X_{1}+0.0081X_{2}+0.053X_{3}-0.098X_{4}-0.0086{X_{1}}^{2}-0.0035{X_{2}}^{2}-0.011{X_{3}}^{2}-0.047{X_{4}}^{2}\\ +0.0005625X_{1}X_{2}+0.002063X_{1}X_{3}+0.000437X_{1}X_{4}+0.00218X_{2}X_{3}+0.006438X_{2}X_{4}+0.014X_{3}X_{4} \end{array}$$

(3) $$\begin{array}{l} \;Y_{3}(\mathrm{mg}/g)=1.17+0.078X_{1}+0.037X_{2}+0.075X_{3}-0.049X_{4}-0.0040{X_{1}}^{2}-0.015{X_{2}}^{2}-0.041{X_{3}}^{2}-0.18{X_{4}}^{2}\\ -0.006125X_{1}X_{2}+0.008X_{1}X_{3}+0.0045X_{1}X_{4}-0.009625X_{2}X_{3}+0.017X_{2}X_{4}+0.054X_{3}X_{4} \end{array}$$

(4) $$\begin{array}{l} Y_{4}(\mathrm{mg}/g)=0.1.18+0.066X_{1}+0.056X_{2}+0.069X_{3}+0.025X_{4}-0.045{X_{1}}^{2}-0.005{X_{2}}^{2}-0.05{X_{3}}^{2}-0.24{X_{4}}^{2}\\ +0.024X_{1}X_{2}+0.019X_{1}X_{3}+0.026X_{1}X_{4}+0.039X_{2}X_{3}-0.011X_{2}X_{4}+0.051X_{3}X_{4} \end{array}$$

where X_1_, X_2_, X_3_ and X_4_ are the coded variables, i.e., extraction time, temperature, pressure and ethanol concentration, respectively.

The reliability of the generated model was evaluated by analysis of variance (ANOVA) and a statistical summary was given in Table 2. *p* value was a measure of the statistical significance, and *R*^2^ represented the proportion of the total variability that had been explained by the mathematical mode [22]. All regression showed that the *p* values were less than the significance level of 0.05, validating adequacy of these models. When *p* value was less than 0.05, the factor had significant impact on the response. Values of *R*^2^ were 0.97 for total alkaloids, 0.99 for peimisine, 0.99 for peimine and 0.98 for peiminine, which illustrated that the models were able to explain variability of 99% in new data for peimisine and peimine combination, and 97% for total alkaloids and peiminine combination. The values of the adjusted determination coefficient were *R*^2^ = 0.95 for total alkaloids, 0.99 for peimisine, 0.99 for peimine, and 0.96 for peiminine.

### 2.3. Effect of the Factors

**Figure 3:**
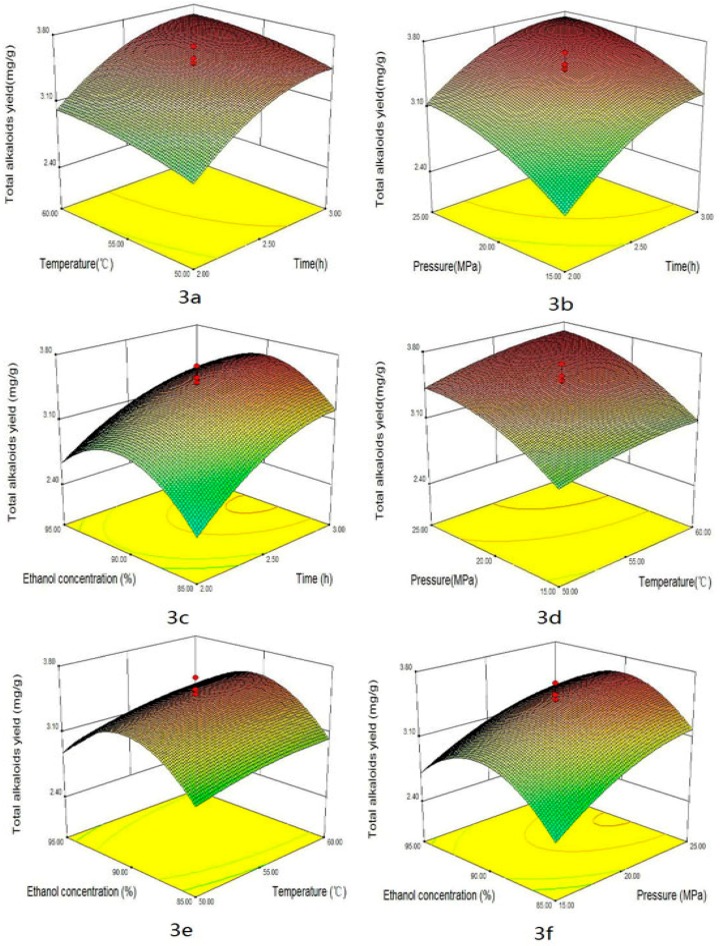
Response surface plots of total alkaloids yields showing: (**a**) the effect of time and temperature at constant pressure (20 MPa) and ethanol concentration (90%); (**b**) the effect of time and pressure at constant temperature (55 °C) and ethanol concentration (90%); (**c**) the effect of time and ethanol concentration at constant temperature (55 °C) and pressure (20 MPa); (**d**) the effect of temperature and pressure at constant time (2.5 h) and ethanol concentration (90%); (**e**) the effect of temperature and ethanol concentration at constant time (2.5 h) and pressure (20 MPa); and (**f**) the effect of pressure and ethanol concentration at constant time (2.5 h) and temperature (55 °C).

**Figure 4:**
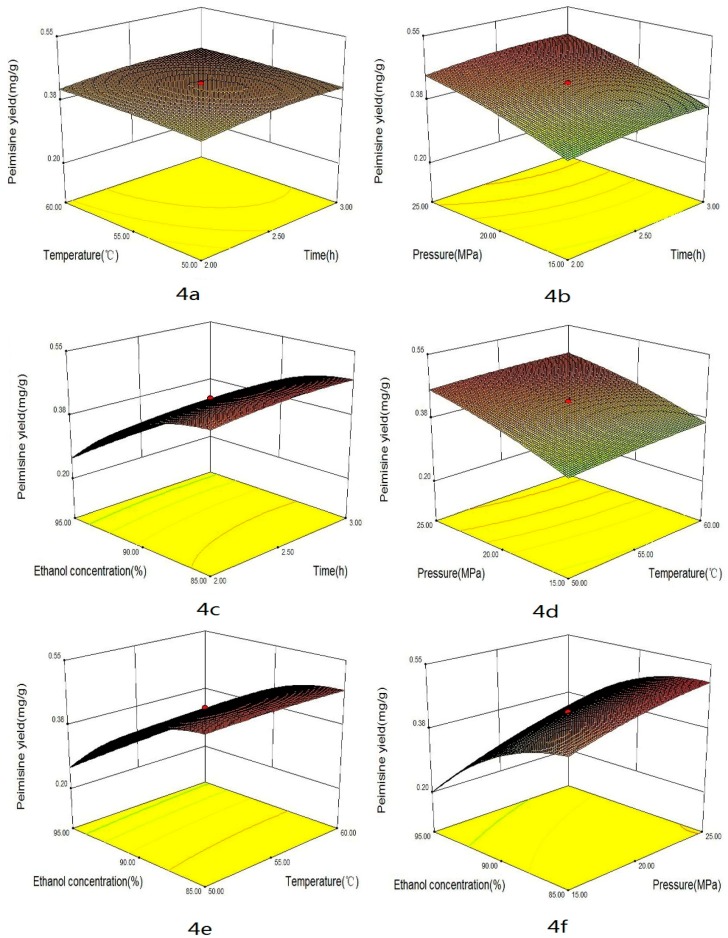
Response surface plots of peimisine yields showing: (**a**) the effect of time and temperature at constant pressure (20 MPa) and ethanol concentration (90%); (**b**) the effect of time and pressure at constant temperature (55 °C) and ethanol concentration (90%); (**c**) the effect of time and ethanol concentration at constant temperature (55 °C) and pressure (20 MPa); (**d**) the effect of temperature and pressure at constant time (2.5 h) and ethanol concentration (90%); (**e**) the effect of temperature and ethanol concentration at constant time (2.5 h) and pressure (20 MPa); and (**f**) the effect of pressure and ethanol concentration at constant time (2.5 h) and temperature (55 °C).

**Figure 5:**
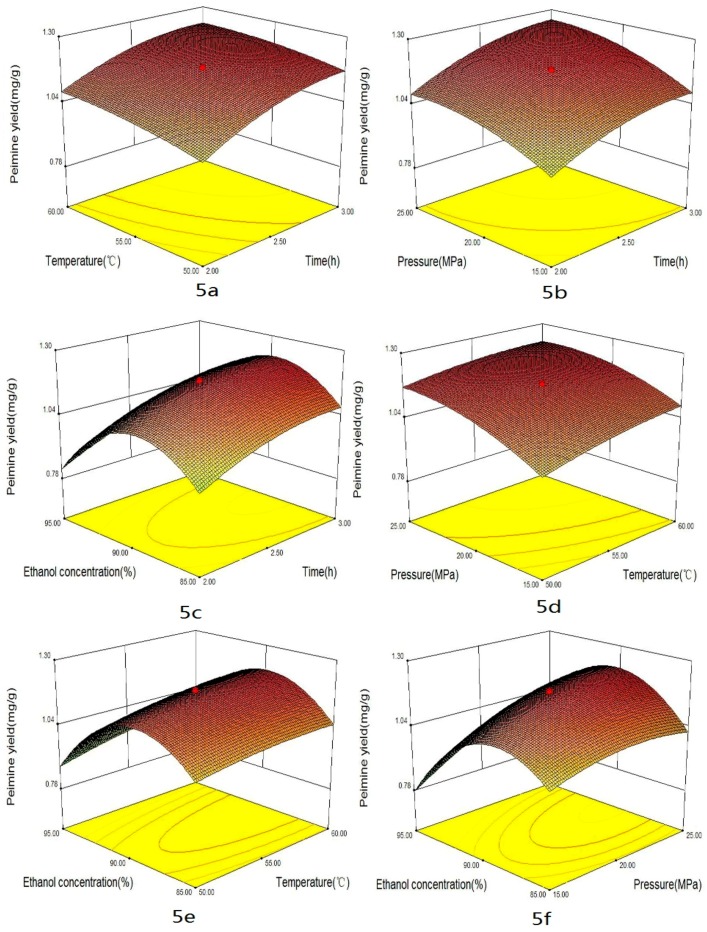
Response surface plots of peimine yields showing: (**a**) the effect of time and temperature at constant pressure (20 MPa) and ethanol concentration (90%); (**b**) the effect of time and pressure at constant temperature (55 °C) and ethanol concentration (90%); (**c**) the effect of time and ethanol concentration at constant temperature (55 °C) and pressure (20 MPa); (**d**) the effect of temperature and pressure at constant time (2.5 h) and ethanol concentration (90%); (**e**) the effect of temperature and ethanol concentration at constant time (2.5 h) and pressure (20 MPa); and (**f**) the effect of pressure and ethanol concentration at constant time (2.5 h) and temperature (55 °C).

**Figure 6:**
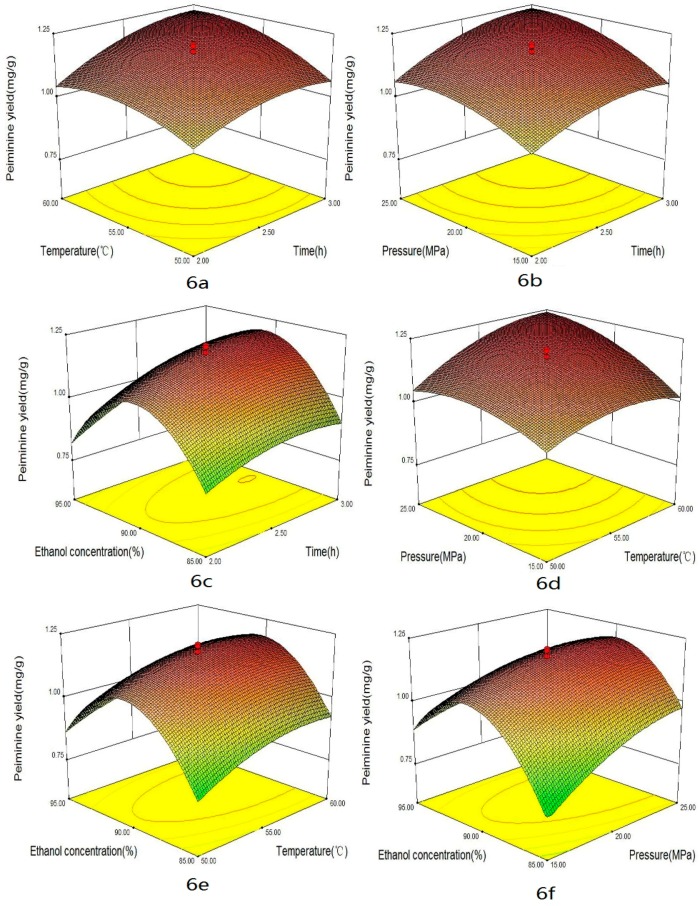
Response surface plots of peiminine yields showing: (**a**) the effect of time and temperature at constant pressure (20 MPa) and ethanol concentration (90%); (**b**) the effect of time and pressure at constant temperature (55 °C) and ethanol concentration (90%); (**c**) the effect of time and ethanol concentration at constant temperature (55 °C) and pressure (20 MPa); (**d**) the effect of temperature and pressure at constant time (2.5 h) and ethanol concentration (90%); (**e**) the effect of temperature and ethanol concentration at constant time (2.5 h) and pressure (20 MPa); and (**f**) the effect of pressure and ethanol concentration at constant time (2.5 h) and temperature (55 °C).

Some factors, such as extraction time, temperature, pressure and co-solvent could impact the yield of alkaloids in supercritical CO_2_ extraction. The combined effects of four factors on the yields of total alkaloids, peimisine, peimine and peiminine are shown in Figure 3, Figure 4, Figure 5 and Figure 6, respectively. These graphs could be used for visually predicting future responses and for determining factor values that optimize the response function. Considering the combination effect of different factors on extraction yield, 3D response surface plots could provide a better understanding of the interaction between any two factors while the other two factors were held at constant optimum values. Figure 3a, Figure 4a, Figure 5a and Figure 6a show the effect of extraction times (X_1_) and temperature (X_2_) on the yields of total alkaloids, peimisine, peimine and peiminine, respectively, while pressure and co-solvent were kept constant at 20 MPa and 90% ethanol. One can see that longer extraction time and higher temperatures favored the extraction. As time increased, the yield increased quickly in the period of time less than 2.5 h, and then the yield increased slightly at time over 2.5 h. This indicated that optimization of extraction time was necessary [12,23]. Shorter time would cause incomplete extraction while longer time would waste time and energy. Higher total alkaloids yield (3.5 mg/g) was achieved at times longer than 2.5 h and temperature higher than 55 °C. In this study, the effect of time was significant for total alkaloids extraction (*p* < 0.05). Moreover, the effect of temperature on the yield could come from two ways. One was the increase of solute vapor pressure with temperature rise to cause an increase of solubility, and another was the decrease of solvent density with temperature rise to cause a decrease of solubility. The improvement of yield depended on which effect was more important. If the effect of vapor pressure were predominant, the solubility of solute in the supercritical phase would increase at higher temperatures, producing higher yield. On the contrary, if the effect of density were overwhelming, the solubility of solute would decrease at higher temperatures. In this study, higher temperature was favor of total alkaloids extraction, which meant that vapor pressure played a major role in the effect of temperature. Temperature had significant effect on the yield (*p* < 0.05). Similar effects of time and temperature on peimisine, peimine and peiminine yields can also be observed in Figure 4a, Figure 5a and Figure 6a, respectively. At times longer than 2.5 h and temperatures over 55 °C, extraction yields of total alkaloids, peimisine, peimine and peiminine were higher than 80%, respectively (all yields being based on the recovery obtained after 3.5 h). The effect of time and temperature was significant for the four interests (*p* < 0.05). However, the interaction effect between time and temperature (X_1_ × X_2_) was not statistically significant (*p* > 0.05) except for the yield of peimine.

The effects of extraction time and pressure (X_2_ × X_3_) on the yields of total alkaloids, peimisine, peimine and peiminine are shown in Figure 3b, Figure 4b, Figure 5b and Figure 6b, respectively. Higher pressure enhanced the extraction efficiency and the higher yields (3.4 mg/g, 0.4 mg/g, 1.2 mg/g and 1.2 mg/g for total alkaloids, peimisine, peimine and peiminine, respectively) were attained at the extraction time of 2.5 h and the pressure of 20 MPa. The phenomenon of higher yield obtained at higher pressure could be attributed to the increase of fluid density with elevating pressure, causing an increase of solubility. The effect of pressure was significant for the four analytes. The interaction between X_1_ and X_3_ was significant for peimine (*p* < 0.05), but not significant for the others.

For extraction of alkaloids with supercritical CO_2_, it was necessary to add a small amount of polar co-solvent in CO_2_ in order to increase the polarity of fluid, so as to improve extraction efficiency and reduce extraction time [12,24,25]. Figure 3c, Figure 4c, Figure 5c and Figure 6c display the effect of extraction time and ethanol concentration (X_1_ × X_4_) on yields of total alkaloids, peimisine, peimine and peiminine, respectively, while temperature and pressure were kept constant at 55 °C and 20 MPa. The yields of total alkaloids, peimine and peiminine were improved from 1.8 mg/g to 3.5 mg/g, from 0.5 mg/g to 1.2 mg/g, and from 0.2 mg/g to 1.1 mg/g, respectively, as ethanol concentration increased from 80% to 90%, and then the yields reduced as the ethanol concentration further increased. For extraction of peimisine, however, the higher yield could be attained by using lower ethanol concentration (80%). The effect of ethanol concentration could be explained by the fact of a similar polar solvent dissolving a similar polar solute. Higher yield could be attained when the polarity of the fluid matched with the polarity of the analytes. It was believed that the solubility of alkaloids increases at a given concentration range of ethanol/water, which resulted in the increase in the extraction recovery [11,26]. Ethanol concentration had significant effect on the yields of the four analytes. X_1_ × X_4_ interaction was significant for peimine (*p* < 0.05), but not significant for the others.

The effects of pressure and temperature on the yields of total alkaloids, peimisine, peimine and peiminine are shown in Figure 3d, Figure 4d, Figure 5d and Figure 6d, respectively. The increase of temperature and pressure enhanced the yields of the four analytes. Higher extraction yields were obtained in temperature between 55 and 65 °C, and pressure between 20 and 30 MPa. X_1_ × X_3_ interaction was significant for peimine and peiminine (*p* < 0.05), but not significant for the alkaloids and peimisine (*p* > 0.05).

The effects of temperature and ethanol concentration interaction (X_2_ × X_4_) on the yields are illustrated in Figure 3e, Figure 4e, Figure 5e and Figure 6e. It could be seen that higher yield was attained in the range of ethanol concentration between 82% and 92%, and temperature between 52 and 65 °C for the extraction of total alkaloids, peimine and peiminine. It was suitable to use <75% ethanol as co-solvent for getting higher peiminine yield (Figure 4e). The interaction X_2_ × X_4_ was significant for peimine (*p* < 0.05), but not significant for total alkaloids peimisine and peiminine (*p* > 0.05).

Figure 3f, Figure 4f, Figure 5f and Figure 6f show the effects of pressure and ethanol concentration (X_3_ × X_4_) on the yields of total alkaloids, peimisine, peimine and peiminine, respectively. The yields were improved as pressure increased for the four analytes at certain ethanol concentration. The yields could be influenced obviously by ethanol concentration. Higher yields of total alkaloids and peiminine appeared in the range of ethanol concentration between 83% and 92%, while higher yield of peimisine was attained at ethanol concentration below 87%. The interaction (X_3_ × X_4_) was significant for the extraction of peimisine, peimine and peiminine (*p* < 0.05). For the total alkaloids, however, the interaction (X_3_ × X_4_) was insignificant (*p* > 0.05).

### 2.4. Antioxidant Activities and Correlation with Total Alkaloids

**Figure 7:**
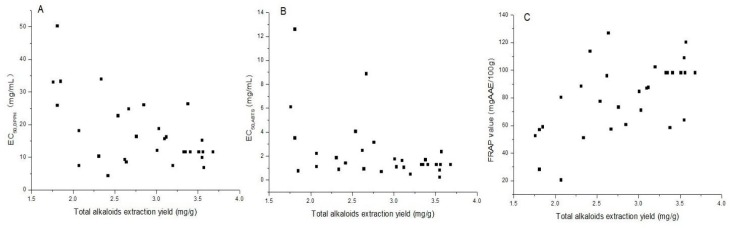
Antioxidant activities and correlation with the extracts of SFE: (**A**) DPPH-RSA; (**B**) ABTS-RSA; and (**C**) FRAP.

The antioxidant activity of *F. thunbergii* bulb was determined by DPPH, ABTS and FRAP methods to give a comprehensive prediction for antioxidant efficacy of the extracts. As shown in Figure 7, the antioxidant activities varied widely and significantly. The antioxidant activity ranged from 4.4 to 50.3 mg/mL by DPPH assay, from 0.2 to 12.6 mg/mL by ABTS assay, and the FRAP value as a measurement of the reducing power ranged from 20.4 to 126.9 mg AAE/100 g for the extracts. The extract could exhibit stronger antioxidant power in vivo because the intervention of enzymes and products caused radicals scavenging and increased the activities of antioxidative enzymes [27,28].

Relevant connection between alkaloids and their antioxidant activity was found in the literature [29,30,31]. In this work, attempts were made to analyze the correlation between the antioxidant activities (DPPH-RSA, ABTS-RSA, and FRAP) and total alkaloids yields using the Pearson’s correlation coefficient (*r*). The correlation coefficient (r) between the total alkaloids and antioxidant capacity of the extracts was −0.55 for DPPH-RSA, −0.23 for ABTS-RSA and 0.58 for FRAP assay. Antioxidant activities as measured by DPPH and FRAP methods showed moderate correlation with the total alkaloids yields, and antioxidant activity as measured by ABTS methods showed low correlation with the total alkaloids yields. These alkaloids compounds were not only significant to the function of relieving cough and reducing sputum, but also to antioxidant power. The different results found by these three methods could be explained by the involved mechanisms. ABTS-RSA and FRAP assays are based on electron transfer (ET) mechanism. In the case of DPPH assay, the reaction between DPPH radicals and the antioxidants could go simultaneously through a HAT (hydrogen atom transfer) and ET mechanisms [32,33]. What is more, antioxidant capacity also depended on pH value, as well supported by several research reports [34,35,36].

### 2.5. Optimization of Extraction Conditions and Verification Tests

The final purpose of determining the levels of key processing variables was to produce an extract with highest alkaloids content. For the four responses, the optimal conditions were obtained using Design Expert software as follow: extraction time, 3.0 h; extraction temperature, 60.4 °C; extraction pressure, 26.5 MPa; and ethanol concentration, 89.3%. As shown in Table 3, the respective experimental values of 3.8 mg/g, 0.5 mg/g, 1.3 mg/g and 1.3 mg/g for total alkaloids, peimisine, peimine and peiminine well matched the predicted values of 3.8 mg/g, 0.5 mg/g, 1.3 mg/g and 1.3 mg/g, respectively. The good agreement between the observed and estimated values verified that the fitted model for each response was reliable to simulate SFE of alkaloids from *F. thunbergii* bulb.

In order to better elucidate the SFE efficiency of alkaloids from *F. thunbergii* bulb, Soxhlet extraction was carried out as a comparison. As seen in Table 3, the yields of total alkaloids, peimine and peiminine obtained by SFE extraction was increased by 30% compared to those by Soxhlet extraction, and the yield of peimisine was particularly increased 67%. EC_50, DPPH_ and EC_50, ABTS_ value of SFE extraction were 0.5 and five times higher than those, and FRAP value of Soxhlet extraction was twice that of SFE extraction, indicating that antioxidant activities of SFE extraction was higher than those of Soxhlet extraction. These results suggested that efficiency of SFE extract was superior to Soxhlet extraction.

## 3. Materials and Methods

### 3.1. Materials and Reagents

The bulbs of *F. thunbergii* were obtained from Zhangshuizhen (Ningbo, China) and dried at 60 °C for 24 h in oven before use. Then, the dried bulbs were ground into powder using an herbal pulverizer (FW 100, Tianjin Taisite Instrument Co. Ltd., Tianjin, China) and sieved under 250 μm size for solvent extraction later. The cylinder of CO_2_ (99.5% purity) were supplied from Fangxin Gas Ltd. (Ningbo, China). Peimine, peiminine and peimisine standards were purchased from the National Institute for the Control of Pharmaceutical and Biological Products (Beijing, China). Acetonitrile of HPLC grade, 2,2-diphenyl-1-picrylhydrazyl (DPPH), 2,2′-azino-bis(3-ethylbenzthiozoline-6) sulfonic acid (ABTS), 2,4,6-tripyridyl-s-triazine (TPTZ), and ascorbic acid were purchased from Sigma Chemical (Louis, MO, USA). Ultrapure water, FeCl_3_6H_2_O, FeSO_4_, Potassium persulfate, sodium acetate, acetic acid, methanol, and ethanol with analytical grade were purchased from Sinopharm Chemical Reagent Co. Ltd. (Shanghai, China). The purities of these compounds were determined to be more than 99.0% by UPLC. Bond Elut-C18 OH columns (500 mg/3 mL) used for solid-phase extraction were purchased from Agilent Technologies (Santa Clara, CA, USA).

### 3.2. Supercritical Fluid Extraction

Supercritical CO_2_ extraction was carried out at Spe-ed SFE-2 (Applied Separation, Hamilton, PA, USA). The extractor volume was 50 mL, thus it was filled with about 15 g of ground bulbs of *F. thunbergii* and the void volume was filled with celite. Flow-rate of CO_2_ (gaseous state) and flow-rate of co-solvent was fixed at 2 L/min and 0.4 mL/min, respectively, during dynamic extraction under each condition. The independent variables included time (1.5, 2, 2.5, 3, and 3.5 h), temperature (45, 50, 55, 60, and 65 °C), pressure (10, 15, 20, 25, and 30 MPa) and co-solvent (ethanol:water) ratio (80%, 85%, 90%, 95%, and 100%, *v*/*v*). After setting the required values according to the experimental design (central composite design), the extracting pressure and temperature were automatically controlled and maintained throughout the system. When both the set pressure and temperature were reached, the extraction was started. At the end of extraction, the extracts were collected from the separator outlet after releasing CO_2_ from the system.

The extracts were quantitatively transferred to a 50 mL volumetric flask by washing the cell with 80% ethanol, in which 3.3 mL were used for measurement of total alkaloids, 2.5 mL used for analysis with UPLC, and the remainder used for activities identification.

### 3.3. Soxhlet Extraction

A certain amount of grounded sample (15.0 g) was accurately weighed and added into a thimble, and then was extracted in a 500 mL of extractor with 375 mL of 89.3% ethanol at a syphon rate of 1 cycle/15 min. After 7 h of extraction, the extraction solvent was essentially colorless and the extracts were transferred to a 500 mL volumetric flask, in which 33 mL were used for measurement of total alkaloids, 2.5 mL used for analysis with UPLC, and the remained used for activities identification.

### 3.4. UPLC-ELSD Analysis

An ultra high performance liquid chromatography system (Agilent, Santa Clara, CA, USA) equipped with an Agilent pump (model L-1290) and an Evaporative Light Scattering Detector (ELSD) detector (Agilent, model L-1260) was used. The column used for separation was a SB-C18 column (1.8 um, 150 mm × 4.6 mm i.d., Agilent Technologies, Beijing, China). The mobile phase was acetonitrile:water:triethylamine (70:30:0.03, *v*/*v*/*v*) at a flow-rate of 1 mL/min. Detection was conducted at a grain of 6, filter of 7 and drift tube was set at 85 °C. For all experiments, 8 μL of standards or sample extract were injected.

The contents of peimisine, peimine and peiminine were determined by referring to the calibration curve established by running standards at varying concentrations through the UPLC system under the same conditions. The calibration curves were linear from 30.0 ug/mL to 600.0 ug/mL (y = −0.91 + 1.52x, *R* = 0.99, *n* = 6, y = Log peak area, x = Log concentration) for peimisine, 22.3 to 1109.6 μg/mL (y = −0.67 + 1.33x, *R* = 0.99, *n* = 6, y = Log peak area, x = Log concentration) for peimine, and from 30.1 to 1666.3 μg/mL (y = −0.88 + 1.41x, *R* = 0.99, *n* = 6, y = Log peak area, x = Log concentration) for peiminine. The limits of detection (LOD) and quantification (LOQ) for each analyte were determined at a signal-to-noise ratio (S/N) of about 3 and 10, respectively. LOD and LOQ of peimisine, peimine and peiminine were 2.0 μg/mL and 11.1 μg/mL, 2.9 μg/mL and 11.4 μg/mL, 3.3 μg/mL and 11.8 μg/mL, respectively.

The intra- and inter-day precisions were evaluated by a standard mixture solution of the three alkaloids under the selected chromatography conditions with five replicates in a day for intraday precision and once a day on three consecutive days for inter-day precision. RSD was taken as a measure of the intra- and inter-day precisions, with 2.0% and 3.1% for peimisine, 2.3% and 3.2% for peimine and 2.1% and 2.8% for peiminine, respectively. Standard addition test was performed to determine recovery in triplicates for each level at two concentrations level. The determined recoveries for peimisine, peimine and peiminine were 98.0%, 99.4% and 101.2% with RSD 3.3%, 3.5% and 4.4%, respectively.

### 3.5. Determination of Total Alkaloids Content

Total content of alkaloids was determined by the Chinese Pharmacopoeia (2010 edition) [7] with some modifications. The extracts were evaporated under reduced pressure at 50 °C to remove the solvent and then the residue was dissolved in 0.5 mL of 0.1 mol/L HCl and 2 mL deionized water. The solution was transferred to a 25 mL volumetric flask, water was added to the flask scale and then filtered. After that, 2 mL of deionized water and 2 mL of bromocresol green were transferred to separating funnel and shaken, and then accurately added 10 mL of CHCl_3_ and vigorously shaken for 2 min. The solution was allowed to stand. Chloroform layer was collected and measured absorbance at 411 nm. According to the standard curve A = 0.04C − 0.01 (A: absorbance, C: peimine concentration, μg/mL, *R* = 0.99, *n* = 6), total alkaloids contents could be calculated.

### 3.6. DPPH Radical Scavenging Assay

The DPPH radical scavenging activity assay was carried out in a Biotek Synergy 2 Multi-Detection Microplate Reader (Biotek, Winooski, VT, USA) according to the procedure described by Chandrasekar et al. [37] with minor modifications. All the experiments were run using a 400 uL 96-well plate. In brief, a series of samples with various concentrations in methanol were prepared, and then 50 uL of each sample solution was mixed with 50 uL of 0.2 mM DPPH solution freshly prepared in methanol. Methanol and l-ascorbic acid were used as the negative and positive control, respectively. After incubation for 30 min at room temperature in the dark, the absorbance of reactant was measured at 517 nm. The measurements of DPPH radical scavenging activity were carried out in triplicate. The percent of radical scavenging activity was determined from the difference in absorbance (A) of DPPH between the negative control and samples.

(5) $$\text{Radical scavenging }(\%)\;=\left[\frac{A_{\text{negative control}}-A_{\text{sample}}}{A_{\text{negative control}}}\right]\times 100$$

### 3.7. ABTS Radical Scavenging Assay

The ABTS radical scavenging assay was performed according to Li’s method [20] with some modification. ABTS radical cation (ABTS•^+^) was produced by reacting ABTS solution (7 mM in water) with 2.5 mM potassium persulfate for 12 h with a ratio of 2:1 (*v*/*v*) at 4 °C in dark (stock solution). Then, the ABTS•^+^ stock solution was diluted with ethanol to an absorbance of approximately 1.0 at 750 nm, which was stable for at least 2 days in the dark. Fifty microliters of diluted ABTS radical solution was mixed with 50 μL of different samples. Ten minutes later, the absorbance was measured at 750 nm against the corresponding blank. The ABTS activity of samples was calculated using the Equation (5).

### 3.8. Ferric Reduction Activity Power (FRAP)

The FRAP assay was performed according to Mário Paz’s method [38]. In short, FRAP reagent (10 mL of 300 mmol/L acetate buffer (pH 3.6), 1 mL of 10 mmol/L TPTZ in 40 mmol/L HCl, and 1 mL of 20 mmol/L FeCl_3_) were diluted to one-third with acetate buffer. One hundred eighty microliters of this solution was added to each well, along with 20 μL of sample. The control assay was performed using 180 μL of FRAP reagent and 20 μL of ethanol. Absorbance was measured at 593 nm and 37 °C. The calibration curve (A = 1.29 × 10^−^^3^C + 0.13, where A: absorbance, C: ascorbic acid concentration, µmol/L, *R* = 0.99, *n* = 6) was prepared with ascorbic acid (AA). The results were expressed as mg ascorbic acid equivalent/100 g (mg AAE/100 g).

### 3.9. Experimental Design and Evaluation

Central composite design (CCD) with four variables and five levels was generated using the Design-Expert Software (Stat-Ease Inc., Minneapolis, MN, USA) in order to optimize the extraction conditions for maximum recovery of bioactive alkaloids and antioxidant capacity. The tested variables and levels were reported in Table 4. In this study, 30 experimental points (16 factorial points, 8 axial points and 6 center points) were conducted to fit a second order polynomial model. The experimental design used for this study was shown in Table 1.

For statistical calculations, the relation between the coded values and actual values were described as the following equation:

(6) $$X_{i}=\left(Z_{i}-Z_{0}\right)/\Delta Z\;i=1,2,3,4,5$$

where *X_i_* was a coded value of the variable; *Z_i_* was the actual value of variable; *Z*_0_ was the actual value of the *Z_i_* at the center point; and Δ*Z* the step change of variable.

The relationship between the response and the independent variables was calculated by the second-order polynomial equation (Equation (7)). The non-linear computer-generated quadratic model was used for this model:

(7) $$Y=\beta_{0}+\sum_{i\;=\;0}^{4}\beta_{i}X_{i}+\sum_{i\;=\;0}^{4}\beta_{ii}{X_{i}}^{2}+\sum_{i}^{3}\sum_{j\;=\;i\;+\;1}^{4}\beta_{ij}X_{i}X_{j}$$

where *Y* was the predicted response; *X_i_* and *X_j_* were independent variables which influenced the response variable *Y*; *β*_0_ was the offset term; *β_i_* was the ith linear coefficient; *β_ii_* was the ith quadratic coefficient; and *β_ij_* was the ijth interaction coefficient.

## 4. Conclusions

In this study, the effects of extraction time, temperature, pressure, and ethanol concentration were evaluated in order to develop an optimized SFE method. The effect of four variables was significant for the extraction of total alkaloids, peimisine, peimine and peiminine (*p* < 0.05). The interaction of pressure and ethanol concentration (X_3_ × X_4_) was significant for the extraction of peimisine, peimine and peiminine (*p* < 0.05). Under the optimal conditions of extraction time 3.0 h, temperature 60.4 °C, pressure 26.5 MPa and ethanol concentration 89.3%, the yields of total alkaloids, peimisine, peimine and peiminine reached 3.8 mg/g, 0.5 mg/g, 1.3 mg/g and 1.3 mg/g, respectively, and they matched with the predicted value very well. From overall analysis, SFE with ethanol as co-solvent could be a useful alternative for the extraction of the compounds with high efficiency. These results proved suitable for the SFE of other alkaloids from other types of plants. Additionally, the extracts of *F. thunbergii* bulb showed good antioxidant activity in vitro, and moderate correlation between total alkaloids yields and antioxidant activity was established. Further studies are essential to evaluate antioxidant activities in vivo and elucidate the antioxidant mechanism.

## Figures and Tables

**Table 1:** CCD experimental design for the yields of total alkaloids, Peimisine, peimine and eiminine, as well as their antioxidant power.

Trial No.	X_1_	X_2_	X_3_	X_4_	Total Alkaloids	Peimisine	Peimine	Peiminine	DPPH	ABTS	FRAP
					mg/g	mg/g	mg/g	mg/g	EC_50_, mg/mL	EC_50_, mg/mL	(mg AAE/100 g)
1	1	−1	1	−1	3.1	0.5	1.0	0.7	16.2	1.1	87.5
2	0	0	0	0	3.4	0.4	1.2	1.2	11.6	1.3	98.2
3	0	0	0	0	3.7	0.4	1.2	1.2	11.6	1.3	98.2
4	0	0	0	2	1.8	0.1	0.3	0.3	33.1	6.1	52.6
5	0	0	0	0	3.3	0.4	1.2	1.1	11.6	1.3	98.2
6	−1	1	1	−1	2.8	0.5	0.9	0.9	16.4	3.2	73.1
7	−1	−1	−1	−1	1.9	0.4	0.8	0.6	33.3	0.8	58.9
8	−1	−1	1	1	2.6	0.3	0.8	0.7	9.3	2.5	95.9
9	−1	−1	1	−1	2.3	0.5	0.9	0.7	34.0	0.9	51.1
10	1	1	1	1	3.2	0.3	1.1	1.0	7.5	0.5	102.3
11	1	1	1	−1	3.4	0.5	1.1	1.1	26.4	1.7	58.4
12	0	0	0	0	3.5	0.4	1.2	1.1	11.6	1.3	98.2
13	−1	1	1	1	2.9	0.3	0.9	0.8	26.0	0.7	60.5
14	1	1	−1	1	3.0	0.2	0.9	0.9	12.1	1.7	84.6
15	0	−2	0	0	3.0	0.4	1.0	0.9	18.8	1.1	71.0
16	1	1	−1	−1	2.7	0.4	1.0	0.7	24.9	8.9	57.2
17	−1	1	−1	−1	1.8	0.4	0.9	0.6	25.9	3.5	56.8
18	1	−1	−1	1	2.5	0.2	0.7	0.8	22.7	4.1	77.3
19	0	0	−2	0	2.4	0.3	0.9	0.9	4.4	1.4	113.8
20	0	0	0	0	3.4	0.4	1.2	1.2	11.6	1.3	98.2
21	−1	−1	−1	1	2.1	0.2	0.6	0.7	18.1	1.1	80.3
22	1	−1	−1	−1	2.6	0.4	1.0	0.6	8.5	0.9	126.9
23	2	0	0	0	3.6	0.4	1.2	1.2	9.9	0.2	109.0
24	−1	1	−1	1	2.3	0.2	0.7	0.7	10.3	1.9	88.4
25	0	0	0	−2	1.8	0.4	0.5	0.2	50.3	12.6	28.2
26	0	2	0	0	3.6	0.4	1.2	1.1	6.9	2.4	120.4
27	1	−1	1	1	3.1	0.3	1.0	0.9	15.7	1.6	87.0
28	0	0	2	0	3.6	0.5	1.2	1.2	15.2	0.8	63.8
29	0	0	0	0	3.6	0.4	1.2	1.2	11.6	1.3	98.2
30	−2	0	0	0	2.1	0.4	0.9	0.9	7.5	2.2	20.4

Note: Experimental values are mean of three determinations.

**Table 2:** Analysis of variance for the response surface quadratic model.

Source	Total Alkaloids			Peimisine			Peimine			Peiminine		
	Sum of Squares	*F*-Value	*p*-Value	Sum of Squares	*F*-Value	*p*-Value	Sum of Squares	*F*-Value	*p*-Value	Sum of Squares	*F*-Value	*p*-Value
Model	10.76	41.50	<0.01	0.37	2147.68	<0.01	1.37	3398.91	<0.01	2.02	50.64	<0.01
X_1_	2.67	144.29	<0.01	2.58 × 10^−3^	211.46	<0.01	0.15	5113.40	<0.01	0.10	36.53	<0.01
X_2_	0.32	17.51	<0.01	1.58 × 10^−3^	129.69	<0.01	0.03	1174.37	<0.01	0.08	26.28	<0.01
X_3_	1.89	101.86	<0.01	0.07	5561.83	<0.01	0.13	4687.03	<0.01	0.11	40.33	<0.01
X_4_	0.04	2.39	0.08	0.23	18,787.21	<0.01	0.057	1998.27	<0.01	0.02	5.27	0.04
X_1_X_2_	6.25 × 10^−6^	6.25 × 10^−6^	0.68	5.06 × 10^−6^	0.41	0.51	6.00 × 10^−4^	20.89	<0.01	9.03 × 10^−3^	3.17	0.09
X_1_X_3_	0.02	1.17	0.26	6.81 × 10^−5^	5.57	0.32	1.02 × 10^−3^	35.63	<0.01	5.63 × 10^−3^	1.98	0.18
X_1_X_4_	0.07	3.72	0.13	3.06 × 10^−6^	0.25	0.87	3.24 × 10^−4^	11.27	<0.01	0.01	3.87	0.06
X_2_X_3_	6.01 × 10^−3^	6.01 × 10^−3^	0.42	7.66 × 10^−5^	6.27	0.16	1.48 × 10^−3^	51.58	<0.01	0.02	8.44	0.01
X_2_X_4_	8.56 × 10^−3^	8.56 × 10^−3^	0.52	6.63 × 10^−4^	54.28	0.10	4.56 × 10^−3^	158.54	<0.01	2.03 × 10^−3^	0.71	0.41
X_3_X_4_	0.04	2.11	0.12	3.05 × 10^−3^	249.87	<0.01	0.05	1593.52	<0.01	0.04	14.76	<0.01
X_1_^2^	0.88	47.36	<0.01	2.05 × 10^−3^	167.42	<0.01	0.04	1501.71	<0.01	0.06	19.87	<0.01
X_2_^2^	0.09	4.70	0.03	3.38 × 10^−4^	27.67	0.93	6.10 × 10^−3^	212.36	<0.01	0.07	24.49	<0.01
X_3_^2^	0.50	27.02	<0.01	3.03 × 10^−3^	248.02	0.03	0.05	1578.38	<0.01	0.07	24.49	<0.01
X_4_^2^	5.19	280.27	<0.01	0.06	4988.22	<0.01	0.93	32,195.41	<0.01	1.60	562.62	<0.01
Residual	0.28			1.83 × 10^−4^			4.31 × 10^−4^			0.04		
Lack of Fit	0.19	1.02	0.52	1.42 × 10^−4^	1.72	0.29	1.99 × 10^−4^	0.43	0.88	0.04	4.41	0.06
Pure Error	0.09	*R*^2^	0.97	4.13 × 10^−5^	*R*^2^	0.99	2.31 × 10^−4^	*R*^2^	0.99	4.35 × 10^−3^	*R*^2^	0.98
Cor total	11.04			0.37			1.37			2.06		

**Table 3:** Extraction yields of total alkaloids, peimisine, peimine and peiminine and antioxidant capacity of total extract from the bulb of *F. thunbergii* Miq. (mean ± SD, *n* = 4).

Extraction Condition	Total Alkaloids Yield (mg/g)		Peimisine Yields (mg/g)		Peimine Yields (mg/g)		Peiminine Yields (mg/g)		DPPH EC_50_(mg/mL)	ABTS EC_50_ (mg/mL)	FRAP (mg AAE/100 g)
**SFE: extraction**	**3.8 ^E^**	**3.8 ^P^**	**0.5 ^E^**	**0.5 ^P^**	**1.3 ^E^**	**1.3 ^P^**	**1.3 ^E^**	**1.3 ^P^**	**5.5**	**0.3**	**118.2**
**Soxhlet extraction**	**3.0**		**0.3**		**1.0**		**1.0**		**8.4**	**1.8**	**58.2**

Note: ^E^, experimental; ^P^, predicted; (SFE extraction condition, time, 3.0 h; temperature, 60.4 °C; pressure, 26.5 MPa; modifier, 89.3% ethanol; flow, 0.4 mL/L; CO_2_, 2 g/L. Soxhlet extraction condition: time, 7 h, solvent, 89.3% ethanol).

**Table 4:** Variables and experimental design levels for response surface.

Independent Variables	Model	Symbol Coded Factor Level				
		−2	−1	0	+1	+2
Extraction time (h)	X_1_	1.5	2	2.5	3	3.5
Extraction temperature (°C)	X_2_	45	50	55	60	65
Extraction pressure (MPa)	X_3_	10	15	20	25	30
Ethanol concentration (%)	X_4_	80	85	90	95	100

## Abbreviations

The following abbreviations are used in this manuscript:

SFE	Supercritical fluid extraction
SC-CO_2_	Supercritical carbon dioxide
DPPH-RSA	DPPH radical scavenging activity
ABTS-RSA	ABTS radical scavenging activity
FRAP	Ferric reducing capacity
CCD	Central composite design
